# Tentacle patterning during *Exaiptasia diaphana* pedal lacerate development differs between symbiotic and aposymbiotic animals

**DOI:** 10.7717/peerj.12770

**Published:** 2022-01-10

**Authors:** Jason S. Presnell, Elizabeth Wirsching, Virginia M. Weis

**Affiliations:** 1 Department of Integrative Biology, Oregon State University, Corvallis, OR, United States of America; 2 Department of Human Genetics, University of Utah, Salt Lake City, UT, United States of America; 3 Department of Biology, Western Washington University, Bellingham, WA, United States of America

**Keywords:** Symbiosis, Aiptasia, Sea anemone, Laceration, Asexual reproduction, Cnidaria, Anthozoa, Hexacorallia, Breviolum, Symbiodiniaceae

## Abstract

*Exaiptasia diaphana*, a tropical sea anemone known as Aiptasia, is a tractable model system for studying the cellular, physiological, and ecological characteristics of cnidarian-dinoflagellate symbiosis. Aiptasia is widely used as a proxy for coral-algal symbiosis, since both Aiptasia and corals form a symbiosis with members of the family Symbiodiniaceae. Laboratory strains of Aiptasia can be maintained in both the symbiotic (Sym) and aposymbiotic (Apo, without algae) states. Apo Aiptasia allow for the study of the influence of symbiosis on different biological processes and how different environmental conditions impact symbiosis. A key feature of Aiptasia is the ease of propagating both Sym and Apo individuals in the laboratory through a process called pedal laceration. In this form of asexual reproduction, small pieces of tissue rip away from the pedal disc of a polyp, then these lacerates eventually develop tentacles and grow into new polyps. While pedal laceration has been described in the past, details of how tentacles are formed or how symbiotic and nutritional state influence this process are lacking. Here we describe the stages of development in both Sym and Apo pedal lacerates. Our results show that Apo lacerates develop tentacles earlier than Sym lacerates, while over the course of 20 days, Sym lacerates end up with a greater number of tentacles. We describe both tentacle and mesentery patterning during lacerate development and show that they form through a single pattern in early stages regardless of symbiotic state. In later stages of development, Apo lacerate tentacles and mesenteries progress through a single pattern, while variable patterns were observed in Sym lacerates. We discuss how Aiptasia lacerate mesentery and tentacle patterning differs from oral disc regeneration and how these patterning events compare to postembryonic development in *Nematostella vectensis*, another widely-used sea anemone model. In addition, we demonstrate that Apo lacerates supplemented with a putative nutrient source developed an intermediate number of tentacles between un-fed Apo and Sym lacerates. Based on these observations, we hypothesize that pedal lacerates progress through two different, putatively nutrient-dependent phases of development. In the early phase, the lacerate, regardless of symbiotic state, preferentially uses or relies on nutrients carried over from the adult polyp. These resources are sufficient for lacerates to develop into a functional polyp. In the late phase of development, continued growth and tentacle formation is supported by nutrients obtained from either symbionts and/or the environment through heterotrophic feeding. Finally, we advocate for the implementation of pedal lacerates as an additional resource in the Aiptasia model system toolkit for studies of cnidarian-dinoflagellate symbiosis.

## Introduction

*Exaiptasia diaphana*, previously *Exaiptasia pallida* (Grajales & Rodríguez, 2014) and commonly called Aiptasia, is a tropical sea anemone that forms a symbiosis with dinoflagellate species in the family Symbiodiniaceae (LaJeunesse et al., 2018). Aiptasia adult polyps are radially symmetric, with several tiers of tentacles arranged around the oral disc and a mouth that leads into a muscular pharynx (Shick, 2012). Running longitudinally along the inner gastrodermal epithelial layer are mesenteries—structures radially arranged around the oral-aboral axis that contain gonads and muscles that help support the body column. The aboral pedal disc is used for locomotion and helps adhere the polyp to surfaces (Shick, 2012; Bedgood et al., 2020; Clarke, Davey & Aldred, 2020).

Aiptasia has routinely been used as a laboratory model for studying the molecular, cellular, and physiological basis of cnidarian-algal symbiosis—especially in the context of coral symbiosis and bleaching (Weis et al., 2008; Davy, Allemand & Weis, 2012; Baumgarten et al., 2015; Weis, 2019). A major advantage of using Aiptasia as a lab model is the ability to maintain adults in the lab without any symbiotic dinoflagellates (Weis et al., 2008; Voolstra, 2013; Matthews et al., 2016). These aposymbiotic (Apo) anemones can be used as a comparison to symbiotic (Sym) anemones to assess the influence of nutritional and symbiotic status on molecular, cellular, physiological and metabolic processes (Lehnert et al., 2014; Carlisle, Murphy & Roark, 2017; Kitchen, Poole & Weis, 2017; Matthews et al., 2017; Rädecker et al., 2018; Sorek et al., 2018). Furthermore, Apo anemones can be re-inoculated with Symbiodiniaceae to understand how colonization is influenced by different symbiont species or under different environmental conditions (Neubauer et al., 2017; Gabay, Weis & Davy, 2018; Parkinson et al., 2018; Matthews et al., 2018; Sproles et al., 2020; Herrera et al., 2021).

**Figure 1:**
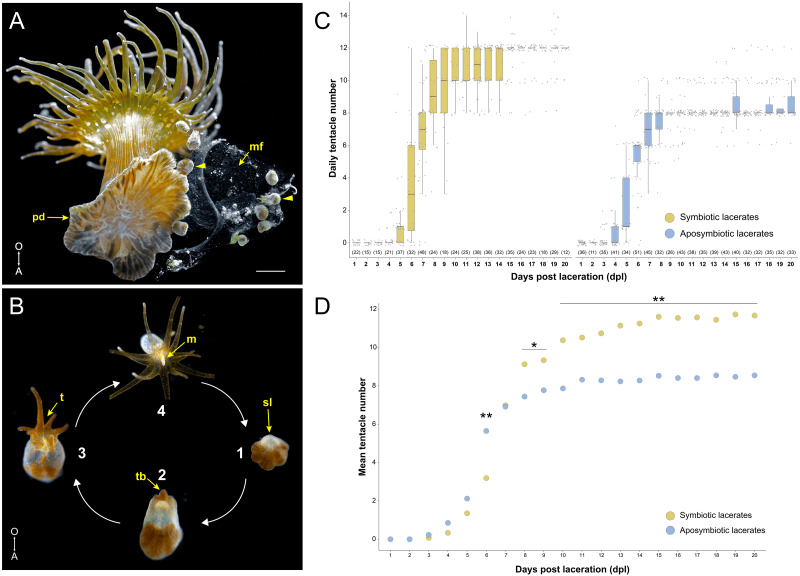
Overview of pedal laceration in *Exaiptasia diaphana*. (A) Aboral view of an adult symbiotic Aiptasia polyp. Pedal lacerates (arrowheads) at different developmental stages can be seen next to the polyp’s pedal disc (pd). Lacerates were typically encased in a “mucus film” (mf). Scale bar = 1.6 mm. Photo credit: Jack C. Koch. (B) Simplified schematic depicting the basic ontogeny of symbiotic pedal lacerates. (1) The site of laceration (sl) heals over and lacks endosymbiotic dinoflagellates while the other tissue remains symbiotic. (2–3) The sl positionally correlates to the oral pole, as the future mouth, oral disc, and tentacle crown will develop on this end of the lacerate. This tissue then extends orally as new tentacles (t) begin to take shape (2–3). (4) Finally, the lacerate reaches a stage where it is essentially a fully formed polyp capable of capturing small prey. After it continues to grow, the newly formed polyp is able to produce lacerates and the cycle continues. Images not to scale. (C) Quantification of pedal lacerate growth as a measure of daily tentacle counts over the course of 20 days for symbiotic (left) and aposymbiotic (right) pedal lacerates. For both plots, center lines show median value, box limits indicate the 25th and 75th percentiles, and whiskers extend 1.5 times the interquartile range from the 25th and 75th percentiles. Numbers of individual lacerates counted are listed underneath each plot. (D) Plot showing mean tentacle counts per day as calculated from data in panel C. *P*-values were calculated using Mann–Whitney *U* test. * = *p* < .05; ** = *p* < .001. For raw numbers of symbiotic and aposymbiotic lacerates, see Data S1. A, aboral pole; O, oral pole; m, mouth; tb, tentacle buds.

Both Sym and Apo Aiptasia laboratory cultures can be rapidly expanded due to their ability to robustly propagate themselves through a process called pedal laceration (Hunter, 1984; Chen, Chang & Soong, 2012; Leal et al., 2012; Armoza-Zvuloni et al., 2014). This form of asexual reproduction has been historically well known to occur in Aiptasia (Cary, 1911; Stephenson, 1929; Smith & Lenhoff, 1976; Hunter, 1984; Clayton Jr, 1985; Clayton & Lasker, 1985; Lin et al., 1992) as well as in other anemone species (Stephenson, 1929; Atoda, 1955). Pedal laceration occurs when several bits of tissue are simultaneously pinched off from the pedal disc, typically in conjunction with the adult polyp traveling across the substrate (Fig. 1A; Cary, 1911; Atoda, 1973; Lin et al., 1992). According to Cary (1911), the laceration site is healed through the rolling in of the epidermal edges surrounding the opening. As development progresses, the lacerates undergo rapid elongation at the site of laceration and proceed through morphogenetic changes to give rise to a small polyp complete with a new oral disc, mouth, pharynx, and tentacles (Fig. 1B; Cary, 1911; Lin et al., 1992; Chen et al., 2016). During pedal laceration, the original mesenteries in the pedal disc from the adult polyp are carried over. However, as the lacerate develops, these original mesenteries atrophy, and are replaced by new lacerate-derived mesenteries in the nascent polyp (Cary, 1911). Lacerates have been shown to develop into polyps within about a week post laceration (Clayton Jr, 1985; Lin et al., 1992; Chen et al., 2016).

Despite these previous descriptions of pedal laceration, the process of how tentacles and mesenteries are patterned throughout development is unknown. Several studies in the non-symbiotic anemone *Nematostella vectensis* have characterized both tentacle and mesentery patterning during different stages of postembryonic development (Genikhovich et al., 2015; He et al., 2018; Ikmi et al., 2020). During metamorphosis from larvae to primary polyp, *Nematostella* mesenteries are the first structures to develop followed by the four primary tentacles (Genikhovich et al., 2015; He et al., 2018). As a young polyp, *Nematostella* tentacle growth is nutrient-dependent and is characterized by sequential, paired emergence around the directive axis in a stereotypical fashion (Ikmi et al., 2020). It is unclear whether nutritional availability has a similar effect on tentacle and/or mesentery growth in Aiptasia lacerates. In addition, even though no difference between Sym and Apo lacerate development time were previously reported (Clayton, 1985), a more thorough examination of how symbiotic state and/or additional nutrients might influence pedal lacerate development and patterning is warranted.

In this study, we compare Aiptasia pedal lacerate development between symbiotic and aposymbiotic individuals. We found differences in the timing of pedal lacerate tentacle formation and we described the patterning events of both tentacles and mesenteries during development. In addition, we demonstrate the potential need for a secondary nutrient source to support continued growth and development of pedal lacerates. Our results suggest that the variation in Aiptasia pedal lacerate growth and development is due to the differences in nutritional supply between aposymbiotic and symbiotic individuals. Finally, we discuss the implications for establishing pedal lacerates as a system to explore mechanisms of symbioses (*e.g.*, algal colonization) and for performing genetic manipulations to dissect specific gene functions in Aiptasia.

## Materials and Methods

### Dinoflagellate and Aiptasia husbandry

Our SSB01 dinoflagellate cultures used in this study are assumed to be either *Breviolum minutum* or a closely related *Breviolum* species (Xiang et al., 2013; LaJeunesse et al., 2018). Sub-cultures of SSB01 were maintained in 50–100 ml of silica-free f/2 liquid medium (Xiang et al., 2013) at 25 °C under 30–40 µmol quanta m-2 s-1 5,000K fluorescent light (Zoo Med Flora Sun) on a 12-hour/12-hour light/dark photoperiod. Mean algal culture density was determined by measuring several aliquots of culture in an automated cell counter (Countessa, ThermoFisher Scientific).

Laboratory stocks of the H2 clonal strain of *Exaiptasia diaphana* (Aiptasia) harboring dinoflagellate symbionts from the genus *Breviolum* (Xiang et al., 2013), were kept in clear plastic containers (Cambro 92CW135) in artificial seawater (ASW; Instant Ocean) at a salinity of ∼32 ppt. The clonal H2 strain was derived from a single individual collected at Coconut Island, HI (Xiang et al., 2013). Symbiotic Aiptasia stocks were maintained at 25 °C under 30–40 µmol quanta m-2 s-1 10,000K fluorescent light (Zoo Med Ocean Sun) on a 12-hour/12-hour light/dark photoperiod. Aposymbiotic Aiptasia stocks were produced following a modified menthol-bleaching method from (Matthews et al., 2016). A 20% w/v stock of menthol (Sigma Aldrich) dissolved in ethanol was diluted in ASW to a 0.11% solution, in which symbiotic Aiptasia were incubated for eight hours daily for three days. This was repeated weekly until Aiptasia were symbiont free as assessed by fluorescent microscopy. Aposymbiotic Aiptasia were maintained at 25 °C in black plastic containers (Cambro 92CW110). Both symbiotic and aposymbiotic Aiptasia were fed 3–5 days per week with live brine shrimp nauplii, and ASW was replaced several hours after feedings.

### Collecting pedal lacerates

Relatively large (>0.5 cm oral disc diameter) individual Aiptasia were placed into soda-lime glass culture dishes (4.5-inch diameter; Carolina Biological, Burlington, NC, USA) at least three days prior to generating pedal lacerates. These polyps were not fed brine shrimp during this time. To collect naturally-formed lacerates, the bottoms of the glass dishes were scrubbed and any lacerates present were removed. The dishes were then checked daily for newly-formed lacerates, which were removed (day 0) and placed into 6-well plates, two per well, filled with 0.2 µm filtered ASW (FSW). To freely generate lacerates, a #10 or #15 sterile scalpel was used to surgically remove 1–4 pieces of pedal disc tissue, ranging 250–500 µm in length, per polyp (day 0). These bits of tissue were placed into 6-well plates, two per well, filled with FSW. Lacerates were incubated at 25 °C under 30–40 µmol quanta m-2 s-1 10,000K fluorescent light on a 12-hour/12-hour light/dark photoperiod. FSW was replaced several times a week, unless otherwise specified (see below). Occasionally, lacerates would become enveloped by a thin, film-like material that was mechanically removed with either forceps or rounded ends of glass capillary tubes. The film would appear within the first several days after laceration, and typically would not return once removed. These films were similar in appearance to the mucus films left behind by adult polyps, and could be playing a role in adhesion (Clarke, Davey & Aldred, 2020).

### Pedal lacerate development

#### Naturally-forming compared to surgically-removed lacerates

Naturally-forming lacerates or ones that were surgically removed from symbiotic adult Aiptasia were collected as described above. Development was tracked by recording the daily number of tentacles of each individual lacerate for 20 days. Mean number of tentacles per day was calculated for both naturally-forming and surgically-removed lacerates. *P*-values for comparisons between the number of daily tentacles for both groups were calculated using a Mann–Whitney *U* test, since the data were not assumed to follow a normal distribution. Data were visualized with RStudio (RStudio Team, 2021). There were very few differences in development between naturally-formed or surgically removed lacerates (Fig. S1). Therefore, to better control for the start day (day 0), we used surgically removed lacerates for all subsequent experiments.

#### Symbiotic compared to aposymbiotic lacerates

Lacerates were surgically removed from both symbiotic and aposymbiotic adult Aiptasia and collected as described above. Development was tracked by recording the daily number of tentacles of each individual lacerate for 20 days. The mean number of tentacles per day was calculated for both symbiotic and aposymbiotic lacerates. *P*-values for comparisons between the number of daily tentacles for both groups were calculated using a Mann–Whitney *U* test, since the data were not assumed to follow a normal distribution. Data were visualized with RStudio (RStudio Team, 2021).

A new batch of lacerates was used to track tentacle and mesentery patterning during development. We established a series of developmental stages reflecting major tentacle patterning events that were consistently observed in Apo and Sym lacerates. Brightfield images were obtained with a Leica M165 FC stereo microscope and Axiocam ERc (ZEISS) and edited for brightness and contrast with FIJI (Schindelin et al., 2012) and Photoshop (Adobe).

#### Aposymbiotic lacerates compared to aposymbiotic lacerates fed homogenized brine shrimp

Lacerates were surgically removed from adult aposymbiotic Aiptasia, collected and stored as described above. Brine shrimp nauplii are too large for the newly developed polyps to capture and ingest, so to simulate heterotrophic feeding, brine shrimp were homogenized before fed to aposymbiotic lacerates. Concentrated brine shrimp nauplii were homogenized in FSW followed by centrifugation for 5 min at max speed to pellet exoskeleton and cellular debris. 50 µl of the supernatant was then added directly over the oral disc or presumptive oral disc tissue. Lacerates were fed on day 4, since this was the earliest we observed a mouth opening. Two separate experiments consisted of different feeding frequencies: (1) once on day 4, or (2) every 3 days starting on day 4. Development was tracked by recording the daily number of tentacles of each individual lacerate for 20 days (Data S1), and the mean number of tentacles per day was calculated. *P*-values for comparisons between the number of daily tentacles for both fed groups to unfed Apo and Sym lacerates were calculated using an ANOVA with a Tukey HSD post-hoc test. Data were visualized with RStudio (RStudio Team, 2021).

#### Aposymbiotic lacerates compared to aposymbiotic lacerates incubated with algal cultures

Lacerates were cut from adult aposymbiotic Aiptasia as described above. Lacerates from the same polyp were divided evenly (as much as possible) among control (aposymbiotic) and experimental (inoculated with algae) groups. SSB01 cultures at densities of 1e^6^ cells/ml or 2e^6^ cells/ml resuspended in FSW were used to inoculate aposymbiotic lacerates either on the same day as laceration (day 0) or 4 days post laceration (dpl). On day 0, algae can enter the gastrodermal cavity through the laceration site, while day 4 was the earliest time point that we observed a mouth opening in Apo individuals. Lacerates were incubated with 4 ml of algae/FSW for 24 h. 2 ml of FSW were then added to each well. After another 24 h (48 h total incubation), the solution in each well was completely replaced with 6 ml of FSW. The control group received the same water changes. Although algal clusters were observed within lacerate tissue, no quantitative measurements were taken to assess the degree to which colonization occurred within lacerates. Development was tracked by recording the daily number of tentacles of each individual lacerate for 20 days. The mean number of tentacles per day was calculated for both aposymbiotic and colonized lacerates. For 1e^6^ algal cells/ml experiments: algae added on day 0, *n* = 9 for control and *n* = 10 for experimental lacerates. For 2e^6^ algal cells/ml experiments: algae added on day 0, *n* = 36 for control and *n* = 23 for experimental lacerates; algae added on day 4, *n* = 10 for control and *n* = 13 for experimental lacerates. *P*-values for pairwise comparisons between the number of daily tentacles for all groups were calculated using a Mann–Whitney *U* test, since the data was not assumed to follow a normal distribution. Data were visualized with RStudio (RStudio Team, 2021).

### Fluorescent staining

At different stages of development, pedal lacerates were immobilized for 15-20 min in a 1:1 volumetric ratio of 7.5% MgCl2 (dissolved in dH2O) and FSW. For some lacerates, the oral disc was dissected using a #15c scalpel blade. In 24- or 6-well plates, whole lacerates or dissected oral discs were fixed in 3.7% formaldehyde/0.2% glutaraldehyde/1x PBS for 1 min at room temperature, then fixed in 3.7% formaldehyde/1x PBS and incubated overnight at 4 °C. After several washes in 1x PBS with 0.2% Triton X-100 (PBT), samples were incubated in PBT with 0.5% BSA overnight at 4 °C. Samples were then incubated in 10 µg/ml Hoechst (Invitrogen) and 0.66 µM Alexa Fluor 488 phalloidin (Invitrogen) overnight at 4 °C. Stained samples were washed with PBS several times then passed through a glycerol-wash series, increasing the glycerol percentage at each step. First, samples were incubated in 50% glycerol (in PBS) overnight at 4  °C. Next, samples were washed twice for 10–15 min in 70% glycerol at room temperature. Finally, samples were washed and incubated in 87% glycerol overnight at 4  °C. The next day, samples were washed once in 87% glycerol, mounted in 87% glycerol on glass slides with No. 1.5 coverslips, and imaged. Samples were stored short-term at 4  °C or at −20 °C long-term before imaging. Z-stacks were acquired with a LSM 780 NLO Confocal Microscope (ZEISS). Separate channels for Hoechst, phalloidin, and chlorophyll autofluorescence were captured with a 405 nm Diode, an Argon, and 633 nm HeNe laser, respectively. Images from each channel were processed for brightness, contrast, and pseudocoloring and merged using FIJI (Schindelin et al., 2012) or Photoshop (Adobe).

## Results

### Symbiotic pedal lacerates develop more tentacles than aposymbiotic pedal lacerates

Initially, we tested whether there were any differences between symbiotic (Sym) and aposymbiotic (Apo) pedal lacerate growth and development. We surgically removed lacerates from both Sym and Apo polyps and counted the number of tentacles per lacerate every 24 h over the course of 20 days (Data S1). Apo lacerates, on average, formed tentacles slightly earlier than Sym lacerates and at 6 days post-laceration (dpl), had developed around twice as many tentacles than Sym lacerates (6 Apo tentacles versus 3 Sym tentacles; Figs. 1C, 1D). Between 6 and 7 dpl, Sym lacerates underwent a rapid expansion in tentacle growth and caught up with Apo lacerates. At 7 dpl, both had developed, on average, around 8 tentacles (Figs. 1C, 1D). Between 10–20 dpl, in a majority of Apo lacerates, tentacle growth plateaued at an average of 8 tentacles (Fig. 1C). In contrast, in most Sym lacerates, tentacle growth plateaued at an average of 12 tentacles (Figs. 1C, 1D). These results show that Sym and Apo pedal lacerates differed in how they develop, specifically in the timing of new tentacle growth over a 20-day period, suggesting that differential nutrient availability or symbiotic state accounts for these observed differences, especially those observed in the later stages (10–20 dpl) of lacerate development.

To determine the role of nutrient availability in tentacle formation, we fed homogenized brine shrimp (HBS) to Apo lacerates at different intervals during development and counted daily tentacle numbers (Fig. S2). Apo lacerates provided with HBS once on day 4 showed no difference in daily tentacle number compared to lacerates provided with HBS every three days starting on day 4. However, daily tentacle numbers for lacerates fed HBS were at intermediate numbers compared to control Apo and Sym lacerates, such that HBS fed lacerates plateaued at an average of 10 tentacles compared to 8 and 12 tentacles for Apo and Sym lacerates, respectively (Fig. S2). This suggests that the lack of tentacle growth in Apo lacerates could be due to a lack of additional nutrients that would normally be available from heterotrophic feeding and/or symbiont-derived photosynthates.

We attempted to test whether the addition of symbionts directly influenced Apo pedal lacerate growth, by counting daily tentacle growth in Apo lacerates incubated with SSB01. Although we observed very few differences in tentacle growth compared to control Apo lacerates (Fig. S3), these experiments produced no clear results. Quantification of algal colonization and levels of photosynthates transferred from symbionts was not performed. Thus, additional experiments will be needed to further distinguish if symbiont-derived nutrients play a role in pedal lacerate development.

### Late stage tentacle growth in Sym lacerates followed three patterns

**Figure 2:**
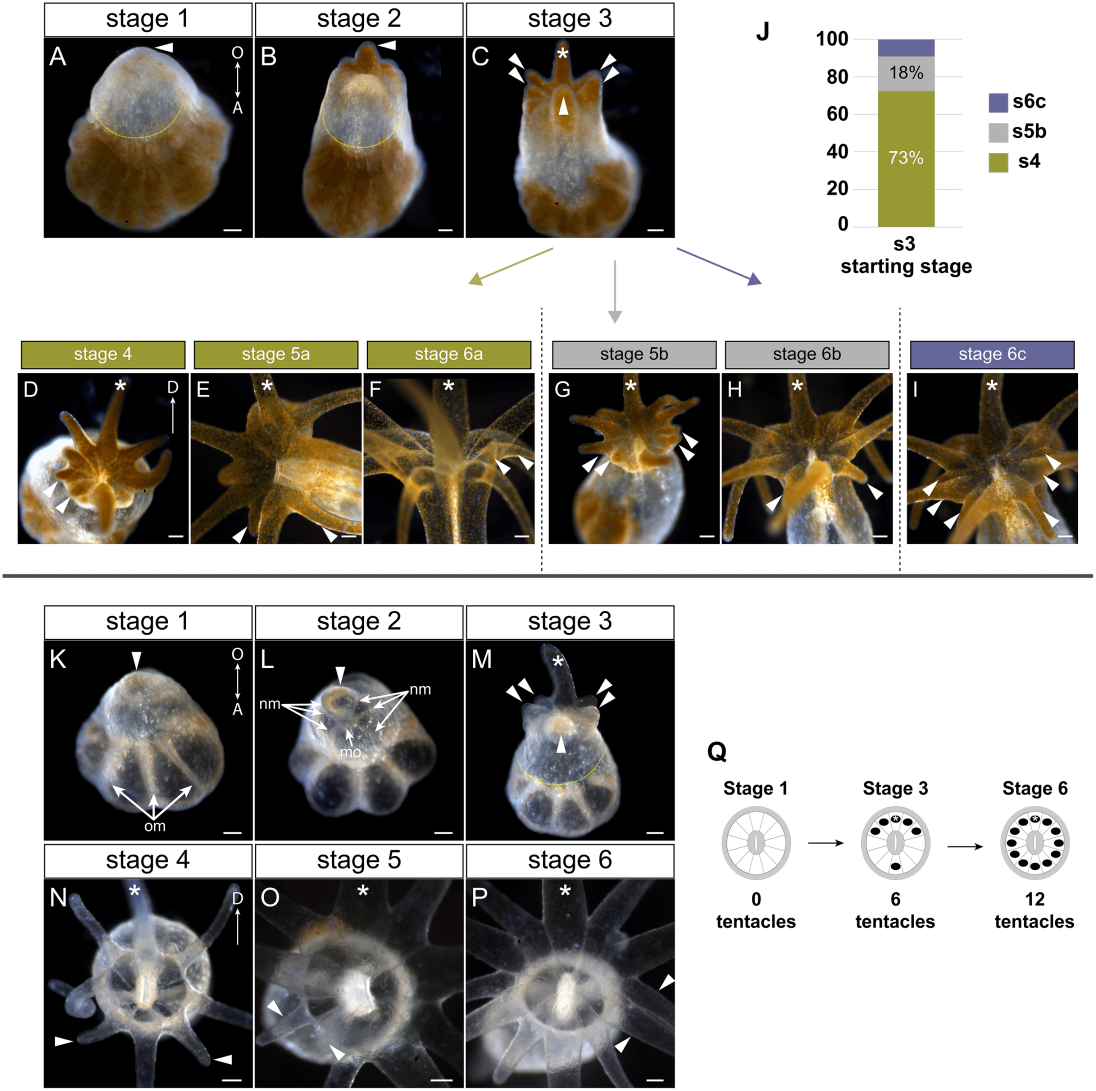
Tentacle patterning during pedal lacerate development. (A–I) Live brightfield images of symbiotic (Sym) pedal lacerates. (K–P) Live brightfield images of aposymbiotic (Apo) pedal lacerates. (A, K) Stage 1 pedal lacerates with primary tentacle bud just starting to show (arrowhead). Original mesenteries (om) carried over from the polyp can be seen in Apo lacerates (K), and algae-free epidermal tissue can be seen extending away from the site of laceration (dotted line) in Sym lacerates (A). (B, L) Stage 2 pedal lacerates with extended primary tentacle (arrowhead) on one side of the nascent oral disc. New mesenteries (nm) are visible in L. We observed a mouth opening (mo) as early as stage 2 (L). (C, M) Stage 3 pedal lacerates with five new tentacles (arrowheads) situated around the primary tentacle (asterisk). (D–I) Oral disc view of Sym lacerates. (D–F) “a” developmental pattern for Sym lacerates following stage 3. New pairs of tentacles (arrowheads) formed at each stage, in a different pattern compared to Apo lacerates. (G, H) “b” developmental pattern for Sym lacerates following stage 3. Four new tentacles (arrowheads) grew simultaneously at stage 5b (G), followed by two additional tentacles at stage 6b (H). (I) “c” developmental pattern for Sym lacerates following stage 3. Lacerates formed six new tentacles simultaneously. (J) Relative percentages of the developmental stage/pattern following stage 3 in Sym lacerates (*n* = 11). (N–P) Oral disc view of Apo lacerates showing new tentacle growth (arrowheads) at stage 4 (N), stage 5 (O), and stage 6 (P). (Q) Schematic showing the pattern of new tentacle growth (black circles) relative to the primary tentacle (asterisk) at stage 1, stage 3, and stage 6. These stages were similar in both Sym and Apo lacerates. Scale bars = 100 µm; O, oral; A, aboral; D, directive axis. *, primary tentacle.

After our initial observations of pedal lacerate development through 20 days, we defined six developmental stages that represented major tentacle patterning events observed in both Apo and Sym lacerates. Each stage corresponds to a specific number of tentacles: stage 1, zero tentacles; stage 2, one tentacle; stage 3, six tentacles; stage 4, eight tentacles; stage 5, ten tentacles; and stage 6, twelve tentacles. We stopped characterizing tentacle patterning at stage 6 (*i.e.*, after 20 days post laceration), since most lacerates remained at the 12- (Syms) or 8- (Apos) tentacle stages for several weeks after 20 dpl (data not shown). Using this staging system, we further characterized lacerate development by describing and comparing the patterning of new tentacle growth between Sym (Figs. 2A–2I) and Apo (Figs. 2K–2P) lacerates.

In both Sym and Apo pedal lacerates, stage 1 occurred between ∼0–3 dpl (Fig. 1D) and was characterized by rapid morphogenesis, which led to wound closure at the site of laceration followed by extension of tissue at this site. Initially, lacerate tissue extended out parallel to the plane of the surface it was adhered to, and only in later stages (stages 3–4), did this tissue turn and grow upwards away from the surface. The distal (oral) end of the extended tissue eventually became the new oral disc and site of new tentacle growth. In Sym lacerates at stage 1, tissue that extended from the site of laceration was mostly free of algae, with a small population inhabiting the future primary tentacle bud (Fig. 2A). At subsequent stages, more algae was observed in the nascent tentacles and oral disc (Figs. 2B, 2C), where it persisted throughout development. Stage 2, occurring at ∼4–5 dpl (Fig. 1D), was characterized by the presence of 1 tentacle which always emerged at the side of the oral disc closest to the surface to which the lacerate was adhered (Figs. 2B, 2L). We designated this tentacle as the primary tentacle and all subsequent descriptions of patterning events are relative to the primary tentacle being at the top in an oral disc view (Fig. 2). Of the lacerates we used for tracking tentacle patterning, both Sym and Apo lacerates, on average, reached developmental stage 3 at roughly the same time, with Apos at 5.43 dpl compared to 6.18 dpl in Syms (Table 1). At this stage, five new tentacles emerged in a consistent pattern observed in both Sym and Apo lacerates. One tentacle was situated directly opposite of the primary tentacle, and the remaining were situated as pairs of tentacles on both adjacent sides of the primary tentacle (Figs. 2C, 2M). The oral disc at stage 3 exhibited bilateral symmetry.

**Table 1:** Mean time to reach developmental stage 3 and stage 6 in Apo and Sym pedal lacerates.

**Symbiotic state**	**N**	**Time to stage 3 (dpl)**		**Time to stage 6 (dpl)**	
		**M**	**SD**	**M**	**SD**
Aposymbiotic	7	5.43	0.79	>20^*a*^	N/A
Symbiotic	11	6.18^*ns*^	1.08	10.00	1.79

**Notes.**

*a*, Apo lacerates did not reach 12 tentacles by 20 dpl. We did not count tentacles after this time point, thus time to reach stage 6 was not calculated for Apo lacerates. dpl, days post laceration. *ns*, not significant. (*p* = .107, Student’s *t*-test); N, number of individuals; M, mean; SD, standard deviation; N/A, not applicable.

Overall, the pattern of tentacles at stages 1, 3, and 6 were the same between Sym and Apo lacerates (Fig. 2Q). In Sym lacerates we observed three different patterns of tentacle growth from stages 3–6, termed the ‘a’, ‘b’, and ‘c’ patterns (Figs. 2D–2J). In the ‘a’ pattern, Sym lacerates progressed through stages 4–6, adding two tentacles at each stage (Figs. 2D–2F). Stage 4a pair of tentacles were situated on the left side of the oral disc adjacent to one of the tentacles added in stage 3 (Fig. 2D). In stage 5a, the new pair of tentacles were located on either adjacent side of the tentacle opposite of the primary tentacle (Fig. 2E) and the stage 6a pair of tentacles were situated on the right side of the oral disc opposite of the tentacles added in stage 4a (Fig. 2F). In the ‘b’ pattern, Sym lacerates skipped stage 4 and progressed through stages 5 and 6 (Figs. 2G, 2H). In stage 5b, four new tentacles were added on either side of the oral disc, adjacent to tentacles added in stage 3 (Fig. 2G). The stage 6b pair of tentacles were situated on either adjacent side of the tentacle opposite of the primary tentacle (Fig. 2H). Finally, in the ‘c’ pattern, Sym lacerates skipped stages 4 and 5, and developed three new tentacles on either side of the oral disc simultaneously (Fig. 2I). 73% of the Sym pedal lacerates we observed developed through the ‘a’ pattern, 18% through the ‘b’ pattern, and 9% through the ‘c’ pattern (Fig. 2J).

In contrast, all Apo lacerates examined progressed through a single pattern from stages 4–6 (Figs. 2N–2P). In stage 4, two tentacles were situated on either adjacent side of the tentacle opposite of the primary tentacle; the oral disc now exhibited radial symmetry (Fig. 2N). The next two pairs of tentacles were added in a bilaterally symmetric fashion. The stage 5 pair of tentacles were situated on the left side of the oral disc between the tentacles added in stages 3 and 4 (Fig. 2O) and the stage 6 pair of tentacles were added in the same position on the opposite (right) side of the oral disc (Fig. 2P). Overall, these results showed that a single pattern in early developmental stages was utilized by pedal lacerates regardless of symbiotic state, and that the differences observed in later Sym lacerate stages were most likely due to the differential nutrient availability between Sym and Apo lacerates.

**Figure 3:**
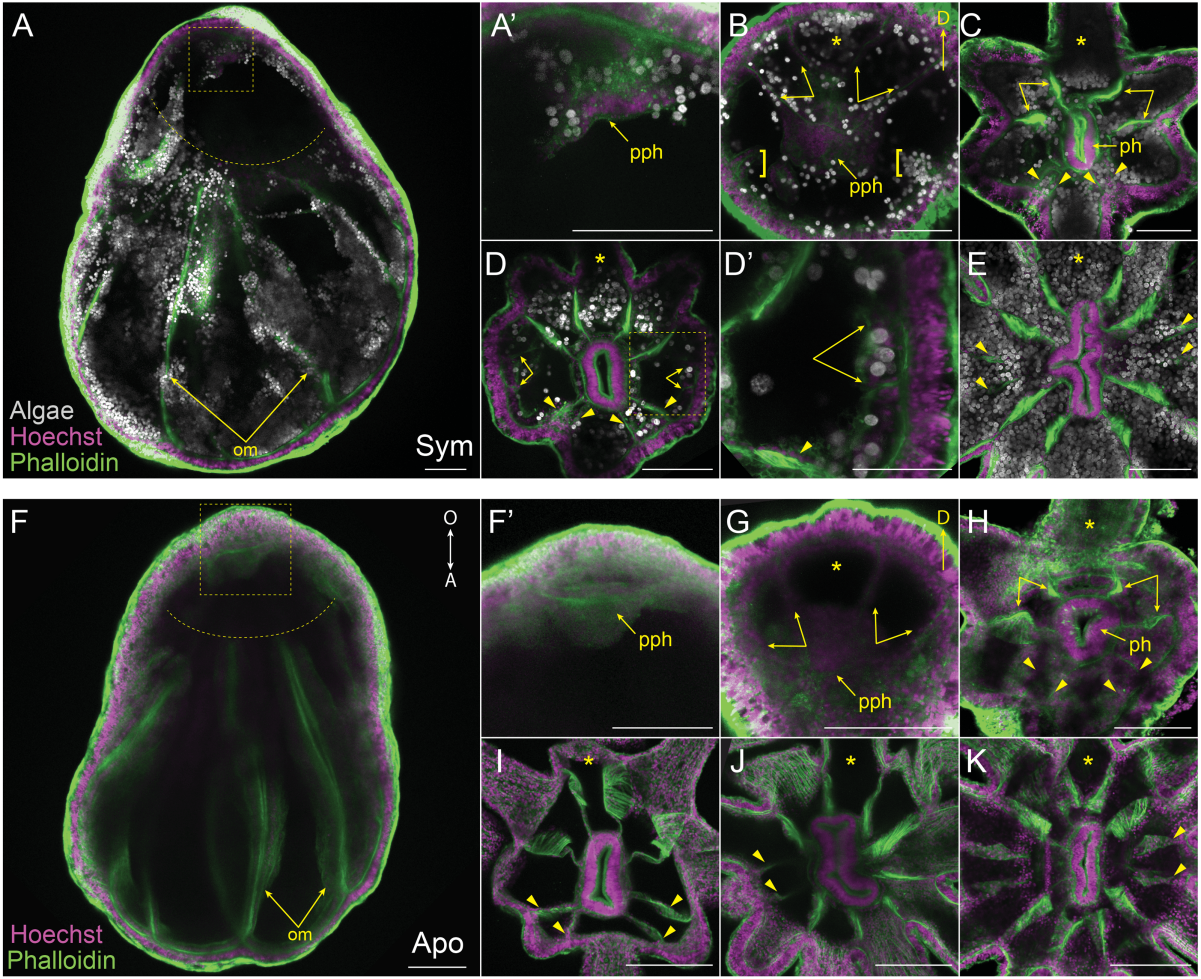
Mesentery patterning during pedal lacerate development. (A–E) Symbiotic (Sym) pedal lacerates stained with Hoechst (magenta) and phalloidin (green); dinoflagellates are visible via chlorophyll autofluorescence (white). (F–K) Aposymbiotic (Apo) pedal lacerates stained with Hoechst (magenta) and phalloidin (green). (A, F) Confocal z-projection of stage 1 pedal lacerate showing that original mesenteries (om) carried over from the polyp did not extend orally past the site of laceration (dotted arc). Box delineates zoomed in region of interest (ROI) in A’ and F’. (A’, F’) Presumptive pharynx (pph) derived from lacerate tissue at the oral end. (B, G) Confocal z-section of stage 2 lacerate oral disc showing the position of the first two pairs of new mesenteries (arrows) relative to the primary tentacle (asterisk) and presumptive pharynx (pph). Brackets indicate mesenteries that have just begun to develop. (C, H) Confocal z-section of stage 3 lacerate oral disc. Arrows indicate the first four mesenteries, arrowheads highlight the second set of four mesenteries to develop. (D) Confocal z-section of stage 4 Sym lacerate oral disc. Arrowheads delineate the second set of four mesenteries to form (see arrowheads in C). At this stage additional mesenteries begin to develop in an atypical pattern (arrows). Box indicates zoomed in ROI in D’. (D’) Gastrodermal compartments formed from new mesenteries (arrows) with resident endosymbiotic algae. (E) Confocal z-section of stage 6 Sym lacerate oral disc showing the newly formed mesenteries (arrowheads). (I–K) Confocal z-sections of stage 4 (I), stage 5 (J), and stage 6 (K) Apo lacerate oral disc showing single pattern of mesentery development relative to the primary tentacle (asterisk). Arrowheads delineate new mesenteries at each stage. Scale bars = 100 µm; O, oral; A, aboral; D, directive axis; *, primary tentacle.

**Figure 4:**
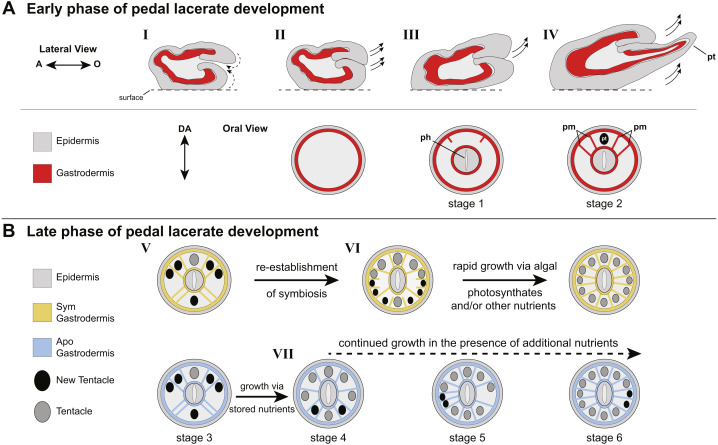
Model of patterning events during Aiptasia pedal lacerate development. (A) Schematic depicting the early phase of lacerate growth and development. Top row: Lateral view, longitudinal section. The aboral end is on the left, the oral end is on the right. Bottom row: Oral disc view, cross section. The directive axis (DA) goes from the top to the bottom. (*I*) Immediately following laceration, the epidermis invaginates into the interior of the lacerate to close the opening at the site of laceration (dotted arrows). (*II–III*) After several days, prior to tentacle growth, tissue at the oral end begins to elongate (arrows) and the new pharyngeal structure (ph) and mesenteries begin to take shape. (*IV*) At stage 2, the lacerate continues to elongate in the oral direction and away from the surface (arrows). The four primary mesenteries (pm) have now developed, two on either side of the primary tentacle (pt). Both Apo and Sym pedal lacerates undergo this early phase of development, where energy is provided to the lacerate from stored nutrients carried over from the adult polyp. (B) Schematic depicting the late phase of pedal lacerate development. Oral disc view, cross section. Top row: Sym lacerates. Bottom row: Apo lacerates. (*V*) Through stage 3, tentacle and mesentery patterning is the same for both Sym and Apo lacerates during development. (*VI*) Sym lacerates undergo a more varied pattern of development after stage 3, in which multiple pairs of mesenteries and tentacles can form in any one of three patterns to reach stage 6. This is most likely due to the abundance of photosynthate-derived nutrients translocated to the host gastrodermal cells from the endosymbiotic algae, once symbiotic homeostasis is reestablished. (*VII*) Apo lacerates undergo a single pattern of development after stage 3 continuing to use nutritional reserves carried over from the adult polyp. At each subsequent stage, a pair of mesenteries forms just prior to a pair of tentacles. A lack of additional nutrients from symbionts and/or the environment causes most Apo lacerates to plateau at eight tentacles; the addition of nutrients should stimulate additional tentacle growth (dashed arrow).

### Pedal lacerate mesentery growth follows a similar pattern to tentacle growth

We aimed to track and characterize new mesentery formation during lacerate development through fluorescent staining of fixed lacerates and confocal microscopy. The F-actin rich mesenteries were easily viewed through fluorescent-phalloidin labeling and confocal microscopy. As seen in Figs. 3A, 3F, the original mesenteries carried over from the adult polyp were severed at the site of laceration and did not continue to grow into the extended lacerate tissue. The first indication of new mesentery growth was an accumulation of tissue localized at the oral end of the developing lacerate (Figs. 3A, 3F’). In Sym lacerates this was one of the first places in the extending oral tissue to contain algal cells (Fig. 3A’). This accumulated tissue eventually gave rise to the pharynx and the first four new mesenteries (Figs. 3B, 3G). In both Sym and Apo lacerates, the first four mesenteries, visible at stage 2, extended from the gastrodermis as two pairs on either adjacent side of the primary tentacle (Figs. 3B, 3G). The next set of mesenteries to form were two pairs situated on the opposite side of the oral disc, mirroring the orientation of the first four mesenteries, for a total of eight (Figs. 3C, 3H, 3I). The arrangement of these mesenteries formed gastrodermal compartments where tentacles would emerge. In stages 1–3, there were more gastrodermal compartments than number of tentacles, such that at stage 3 both Sym and Apo lacerates contained eight mesenteries but only six tentacles (Figs. 3C, 3H). However, by stage 4 in Apo lacerates, the number of tentacles matched the number of mesenteries (Fig. 3I). After the formation of the first eight mesenteries, the remaining four mesenteries developed approximately at the same time in Sym lacerates (Figs. 3D, 3D’) for a total of 12 mesenteries at stage 6 (Fig. 3E). Following stage 4 in Apo lacerates, a pair of mesenteries was added on either side of the oral disc, forming gastrodermal compartments from where the two new tentacles would emerge in stage 5 (Fig. 3J) and, subsequently, stage 6 (Fig. 3K). These results show that, similar to tentacle patterning, mesentery formation follows a single pattern in early developmental stages of Sym and Apo lacerates. In later developmental stages, Apo lacerate mesenteries progressed through a single pattern that mirrored late stage tentacle patterning. Later stages of Sym lacerate mesentery growth were similar to late stage tentacle formation, characterized by variable patterns.

## Discussion

### Pedal lacerate development proceeds through two nutritionally- dependent phases

Overall, our data suggest two different nutritional phases of pedal lacerate development: an early phase where lacerates preferentially use (Sym) or are dependent on (Apos) nutrients carried over from the adult polyp, and a late phase where lacerates are more dependent on “new” nutrients coming from the environment and/or, in the case of Sym lacerates, from their symbionts (Fig. 4). In our initial Sym vs Apo experiments, lacerates were not provided with any additional food or nutritional sources. Thus, Apo lacerates were essentially starved over the course of the 20-day developmental period. However, because Apo lacerates were able to develop into functional polyps, complete with tentacles, a mouth, and pharynx, nutrients were most likely carried over from the parent polyp (Fig. 4B). These resources were sufficient for the lacerates to reach a stage where they could potentially obtain additional nutrients from the environment through acquisition of symbiotic algae and/or through heterotrophic feeding. Previous work has shown that Aiptasia is capable of generating lacerates when only infrequently fed heterotrophically, for example just once every four weeks (Clayton & Lasker, 1985). Several anemone species, including Aiptasia, will initiate asexual reproduction (*e.g.*, pedal laceration) when starved (Smith & Lenhoff, 1976; Sebens, 1980; Clayton Jr, 1985; Leal et al., 2012), which suggests that stored nutrients are utilized by the adult and its clonal propagules until a food source is made available. In anemones, nutrient (sugars, lipids, and oligopeptides) storage occurs in the gastrodermal/endodermal component of complete mesenteries (Steinmetz et al., 2017), and thus the carry-over of mesenteries from adult polyps to lacerates is most likely the source for these nutrients used for the early phase of development, especially in Apo lacerates (Fig. 4B).

The cnidarian-dinoflagellate symbiosis is centered around nutrient exchange—host cells provide dissolved inorganic carbon and nitrogen used for photosynthesis and growth, while algal cells translocate reduced organic compounds (*e.g.*, glucose, fatty acids, lipids) that are utilized by the host to support biological processes such cellular respiration and tissue growth (Muscatine, 1990; Davy, Allemand & Weis, 2012). Although we observed increased growth rate (*i.e.*, tentacle formation) and an overall increase in size during later stages of development in Sym lacerates compared to Apo lacerates, prior to 7 dpl, Sym and Apo lacerates developed at approximately the same rate, with Apo lacerates even forming more tentacles faster over these early stages. This observation seems counterintuitive, since we would have expected that if symbiont-derived nutrients were available to Sym lacerates starting at day 0, development and growth rate would have been consistently higher at all stages in Sym lacerates compared to Apo lacerates. A possible explanation of the lack of rapid growth in the early phase of Sym lacerate development could be due to the interactions between adult-derived nutrients and those derived from resident symbionts. It has been shown in coral larvae that obtain symbionts from the surrounding environment (*i.e.*, horizontally acquired), that the energy from algal photosynthate did not offset the use of egg-provisioned nutrients derived from the parent and that the presence of symbionts provided either a neutral or negative consequence to coral larvae (Hartmann et al., 2018). It is possible that during the early growth phase, Sym pedal lacerates are preferentially utilizing parent-derived nutrients as they work to regain homeostasis with their symbiotic partner. Once homeostasis is established, the host could then utilize the additional nutrients provided by the symbiont to support new tentacle growth and development (Fig. 4B). For example, Sym lacerates continued to develop tentacles and grow in size after 20 dpl without additional, external food sources.

Another explanation for why Sym lacerates did not develop at a faster rate than Apo lacerates in the early stages could lie in the cellular mechanisms underlying lacerate development and new tentacle growth. Initially, very few algae were observed in the oral end of developing lacerates. As seen in Figs. 2A–2C and 3A–3D, the algal population increase was concomitant with oral disc and new tentacle formation. It is possible that as more gastrodermal cells underwent differentiation, more algae were able to invade these newly-formed gastrodermal cells, and additional photosynthates were then provided to the host to support continued growth and development observed in the late stage Sym lacerates. This localized increase in nutrients could have caused an increase in cell proliferation, similar to what has been shown during algal recolonization of adult Aiptasia where the presence of algae in host tissue causes high rates of localized cell proliferation in the gastrodermis housing the algae (Tivey, Parkinson & Weis, 2020). In addition, it has been shown that aposymbiotic Aiptasia polyps have a different circadian rhythmicity than symbiotic polyps (12-hr in Apo, 24-hr in Sym) (Sorek et al., 2018). This could be another explanation of the delayed growth in Sym lacerate early development stages.

Although our data point to a potential role of symbiotic state in mediating pedal lacerate growth, additional experiments are needed to fully disentangle how much of lacerate development is regulated by alga-provided photosynthates or just nutrients in general, such as those acquired from heterotrophic feeding. For example, it would be expected that photosynthesis inhibition, by incubating lacerates in the dark and/or in the chemical inhibitor 3-(3,4-dichlorophenyl)-1,1-dimethylurea (DCMU), would reduce the amount of nutrients provided to Sym lacerates from their symbionts, and thus late-stage development would proceed at a rate similar to Apo lacerates. Another possible experiment would be to generate lacerates from symbiotic adults colonized with different species of algae, both those native to Aiptasia and non-native species. It has been shown that different species of Symbiodiniaceae colonize Aiptasia at different rates and provide different levels of nutrients to Aiptasia hosts (Matthews et al., 2017; Gabay, Weis & Davy, 2018)—it would be expected that a poor symbiotic species would provide Sym lacerates with fewer nutrients, and they would then develop at rates similar to those of Apo lacerates.

After 7 dpl, Sym lacerate growth increased dramatically and patterning of both tentacles and mesenteries was highly variable compared to Apo lacerates. These observed differences between Sym and Apo lacerates could also be explained by a lack of nutrients in general in Apo lacerates, not necessarily those specifically derived from symbionts. In the non-symbiotic anemone *Nematostella vectensis*, new tentacle growth in primary polyps is supported by localized nutrient-dependent cell proliferation (Ikmi et al., 2020). In the absence of food sources (*i.e.*, nutrients) young *N. vectensis* polyps arrest their tentacle growth and remain at the stage of development they were at when starved (Ikmi et al., 2020). The slow-down in overall growth and new tentacle formation in Apo lacerates after ∼7 dpl could be due to a similar mechanism that occurs in starved *N. vectensis* polyps. In support of this, when provided with a potential source of nutrients in the form of homogenized brine shrimp, we demonstrated that Apo lacerates increased their tentacle growth compared to control Apo lacerates (Fig. S2). However, these fed Apo lacerates did not increase their tentacle growth to the level of growth observed in Sym lacerates, suggesting that Sym lacerates had access to additional nutrients, in the form of algal provided photosynthates. We would expect that subjecting adult polyps to different nutritional states would result in lacerates that had variable nutrient profiles at the start of development. Coupled with experiments testing nutrients coming from symbionts (*e.g.*, by photosynthesis inhibition), these experiments would provide additional insights into which source of nutrients, either heterotrophically- or symbiont- acquired, and how much of each source contributes to pedal lacerate development.

### Pedal lacerate development—a unique form of asexual reproduction?

In both Apo and Sym lacerates, we observed sequential, paired formation of mesenteries and tentacles (Fig. 4) suggestive of an axial code that establishes the patterning of lacerate radial structures (mesenteries and tentacles). In addition, the appearance of the primary tentacle in the same spot relative to the oral disc also suggests some inherent genetic network that mediates the identity of specific oral disc regions. In *N. vectensis,* the formation and patterning of both mesenteries and tentacles has been thoroughly described in planula larvae and primary polyps (He et al., 2018; Ikmi et al., 2020). In planula larvae, eight endodermal segment boundaries, that give rise to mesenteries, are patterned in a radial formation around the directive axis (He et al., 2018). During larval development, these endodermal segment boundaries arise as paired structures, one on either side of the directive axis, and are demarcated by Hox-Gbx expression domains (Leclère & Rentzsch, 2014; Genikhovich et al., 2015; He et al., 2018). Tentacle and mesentery patterning in lacerates is morphologically similar to how these structures are patterned and develop in *N. vectensis*. However, one major difference is mesenteries in Aiptasia lacerates were formed sequentially just prior to the formation of their neighboring tentacles, such that development proceeded in a “mesentery-then-tentacle” pattern while in *N. vectensis* all eight mesenteries formed first followed by growth of new tentacles.

In *N. vectensis* polyps, four primary tentacles initially emerge, followed by sequential addition of new tentacle pairs in a nutrient-dependent manner (Ikmi et al., 2020). Each tentacle of a pair emerges on either side of the directive axis (referred to as *trans*-budding) through the 12-tentacle stage, followed by two rounds of *cis*-budding (tentacles on same side of directive axis) to reach the 16-tentacle stage (Ikmi et al., 2020). Both *trans-* and *cis-* budding were observed during Aiptasia lacerate development in both Apo and Sym individuals, though neither Apo or Sym patterning followed the exact tentacle pattern in *N. vectensis*. Overall, while both Apo and Sym pedal lacerate mesentery and tentacle patterning shared some superficial similarities with what has been described for *N. vectensis* patterning (*e.g.*, sequential, paired formation radially around the directive axis), differences were observed between the two species including the “mesentery-then-tentacle” pattern of development seen in Aiptasia.

During planarian regeneration, a tail piece that has been amputated will form the full suite of head and trunk organs and tissues *de novo* (see Ivankovic et al., 2019; Stückemann et al., 2017). This ability for the correct formation of missing body parts during regeneration is due to pre-existing polarity along the anterior-posterior axis of the animal generated through antagonistic signaling gradients (Ivankovic et al., 2019; Stückemann et al., 2017). Our results showing that initial morphogenesis in the early stages of pedal lacerate development always occurred at the site of laceration which became the future oral pole of the new polyp, is suggestive of an axial polarity present in lacerate tissue that defines the future oral and aboral poles. Alternatively, polarity could be re-established after laceration, such that signals from the laceration site turn-on the genetic network that underlies oral-aboral polarity. Overall, our data is suggestive of a genetic network that establishes a pre-existing axial code that underlies the correct patterning and orientation of new structures, and an oral-aboral polarity that enables the correct tissues (*e.g.*, oral disc) to form in the correct position.

In addition, Aiptasia pedal lacerate development is different from the process of oral disc regeneration during which the structures that were present when the animal was amputated (mesenteries, pharynx, tentacles) all emerge and grow back simultaneously, as seen in other anemones (Fig. S4 ; Reitzel et al., 2007; Passamaneck & Martindale, 2012; Amiel et al., 2015; Van der Burg et al., 2020). Overall, Aiptasia pedal lacerate development shares characteristics with *N. vectensis* primary polyp development and planarian head regeneration, but is a separate process from cnidarian oral disc regeneration. Other anemones are known to undergo pedal laceration (Stephenson, 1929; Atoda, 1955; Atoda, 1973), and, thus, comparative studies in these anemones and other anthozoans would help determine if pedal lacerate development, as described in this study, is a unique mode of asexual reproduction found in Aiptasia or conserved, on some level, across Actiniaria or even Anthozoa.

### Pedal lacerates as an additional resource in the Aiptasia- Symbiodiniaceae model system

Aiptasia is a commonly used laboratory model for the study of cnidarian-dinoflagellate symbiosis—typically as a proxy for coral-algal symbiosis (Weis et al., 2008; Rädecker et al., 2018; Weis, 2019). One of the last frontiers for this community of scientists is developing the ability to genetically manipulate the algal and cnidarian genomes to investigate specific gene functions underlying colonization, symbiosis maintenance, and dysbiosis. While pioneering advances have been made in both corals and Aiptasia in targeted genetic manipulation in embryos (Grawunder et al., 2015; Yasuoka, Shinzato & Satoh, 2016; Cleves et al., 2018; Cleves et al., 2020; Jones et al., 2018), technical limitations still persist. For example, while the first CRISPR/Cas9 mutagenesis studies in corals were recently published (Cleves et al., 2018; Cleves et al., 2020), corals can be difficult to keep in laboratory culture and spawning for most coral species is highly infrequent (Weis et al., 2008). While genetic manipulation has not currently been published in Aiptasia, techniques for these types of experiments (embryo microinjection, transgenesis) have been described (Jones et al., 2018). Currently, the biggest hurdle moving forward is the inability to get Aiptasia larvae to settle and undergo metamorphosis in the lab. While the use of F0 embryos is a valid route for generating and testing hypotheses regarding gene function, producing individuals from F1 and subsequent generations that harbor stable, heritable non-lethal mutations and transgenes is most likely superior for characterizing gene functions in a complex process such as symbiosis. While efforts are being made to solve the settlement and metamorphosis problem, in the meantime, we advocate the use of pedal lacerates as a system for developing molecular genetic techniques in Aiptasia. The size of the lacerates allows for relatively easy manipulation via microinjection and/or electroporation, as some preliminary experiments with both methods have successfully delivered fluorescent dyes into both Sym and Apo lacerates (J. Presnell, 2019, unpublished data). In addition, the rapid growth and regenerative ability of lacerates could be leveraged to rapidly reduce mosaicism and generate mutant adult tissue adequate for experimentation. The relatively small size of lacerates allows for straightforward high-resolution imaging which could be utilized for studying the cellular and physiological aspects of symbiosis, such as algal colonization and stress response. Lacerates would offer a compromise between the use of embryos, which are aposymbiotic throughout most of their natural life cycle, and the use of large adult symbiotic polyps which can be unwieldy for high-resolution imaging. Overall, Aiptasia pedal lacerates are an untapped resource to better understand the intersection of biological processes (*e.g.*, development and symbiosis) and to further explore the genetic, cellular, and physiological basis of cnidarian-dinoflagellate symbiosis.

## Conclusions

Aiptasia readily undergo asexual reproduction in the laboratory via the process of pedal laceration. While this process has been documented in the past, details about how the lacerates develop into polyps was less understood. Here we described the growth patterns of tentacles and mesenteries in both symbiotic and aposymbiotic lacerates during their development into polyps. Our results support two phases of lacerate development: an early phase dependent on stored nutrients, and a late phase preferentially utilizing additional nutrients from the environment. In addition, we propose that this foundational descriptive work on pedal lacerates will spur other laboratories to implement this part of Aiptasia, already an extensively used model system, as a component for studying cnidarian-dinoflagellate symbiosis.

## Supplemental Information

10.7717/peerj.12770/supp-1 Supplemental Information 1 Quantification of daily tentacle counts in naturally forming and surgically removed symbiotic lacerates over the course of 20 days Center lines show median value, box limits indicate the 25th and 75th percentiles as determined by R software, and whiskers extend 1.5 times the interquartile range from the 25th and 75th percentiles. *P*-values were calculated using a Mann–Whitney *U* test. Significant *p*-values, *p* < .05, are indicated in bold font (dpl 6 and dpl 20). Raw data is available in Data S1.

10.7717/peerj.12770/supp-2 Supplemental Information 2 Quantification of daily tentacle counts in aposymbiotic lacerates fed homogenized brine shrimp once on day 4, or every 3 days starting on day 4, over the course of 20 days Center lines whose median values, box limits indicate the 25th and 75th percentiles as determined by R software, and whiskers extend 1.5 times the interquartile range from the 25th and 75th percentiles. *P*-values were calculated using a Kruskal–Wallis with post-hoc Tukey HSD test. Significant *p*-values, *p* < .05, are indicated in bold font. Data is unavailable for lacerates fed once on day 4 for dpl 8 and dpl 9. Raw data is available in Data S1.

10.7717/peerj.12770/supp-3 Supplemental Information 3 Quantification of daily tentacle counts in aposymbiotic lacerates incubated with algae over the course of 20 days (A) Apo lacerates incubated with 1 million algal cells/ml starting on day 0, compared to Apo control lacerates. (B) Apo lacerates incubated with 2 million algal cells/ml starting on day 0, compared to Apo control lacerates. (C) Apo lacerates incubated with 2 million/algal cell/ml starting on day 4, compared to Apo control lacerates. Center lines show median value, box limits indicate the 25th and 75th percentiles as determined by R software, and whiskers extend 1.5 times the interquartile range from the 25th and 75th percentiles. *P*-values were calculated using a Mann–Whitney *U* test. ∗ = *p* < .05.

10.7717/peerj.12770/supp-4 Supplemental Information 4 Oral disc regeneration in Aiptasia A young polyp 48 h after oral disc amputation. All structures that were present at the time of amputation, oral disc, pharynx, mesenteries, and tentacles (arrows) grow back simultaneously. Scale bar = 250 µm.

10.7717/peerj.12770/supp-5 Supplemental Information 5 Raw data and statistical tests for each experiment performed

## Additional Information and Declarations

### Data Availability

The following information was supplied regarding data availability: The raw measurements are available in the Supplementary File.
