# Supplementary figures and images for: Tentacle patterning during Exaiptasia diaphana pedal lacerate development differs between symbiotic and aposymbiotic animals
Source: PeerJ. 2022 Jan 10;10:e12770. doi: 10.7717/peerj.12770 (PMC8757374; doi:10.7717/peerj.12770)

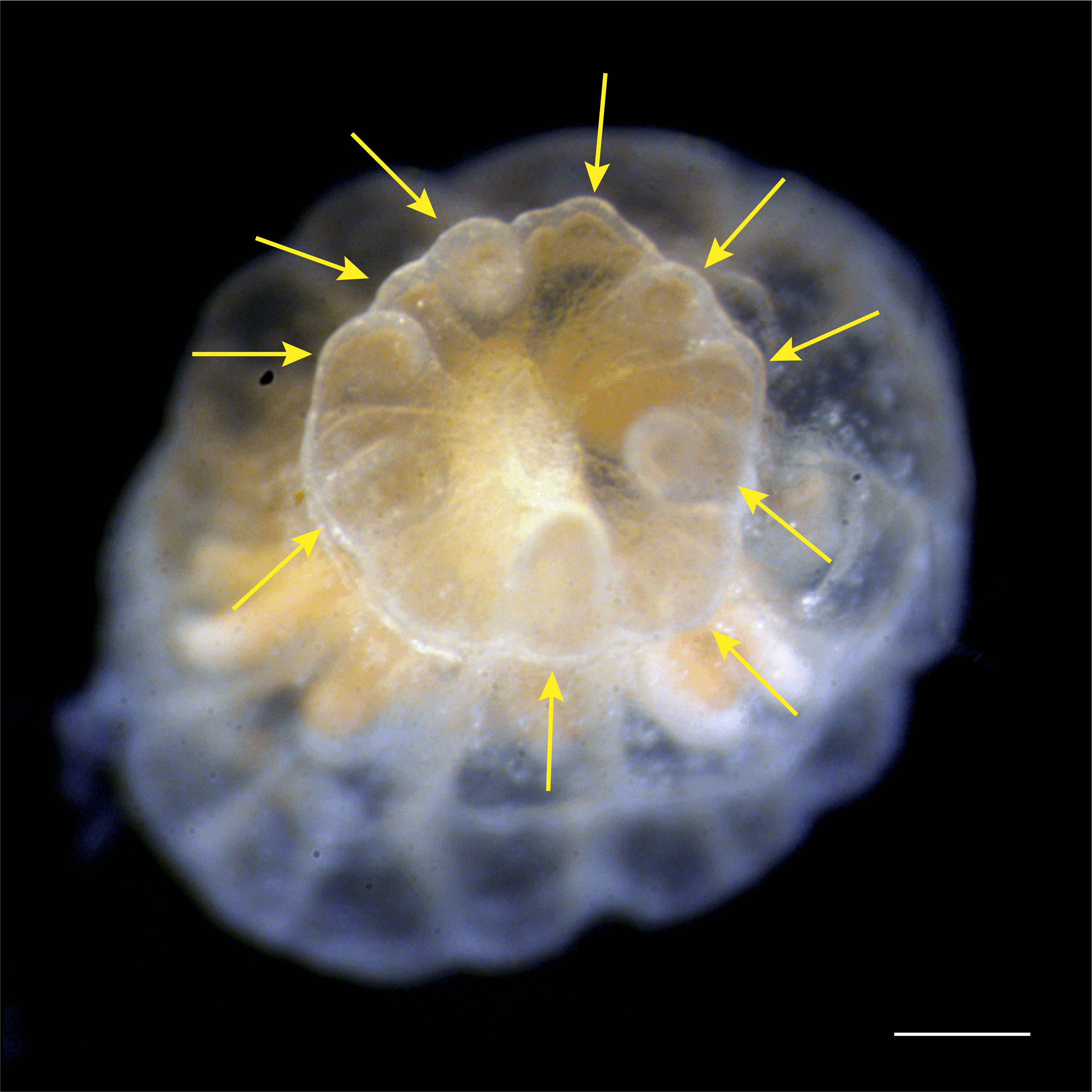

Supplement: Supplemental Information 4 — A young polyp 48 h after oral disc amputation. All structures that were present at the time of amputation, oral disc, pharynx, mesenteries, and tentacles (arrows) grow back simultaneously. Scale bar = 250 µm. [file peerj-10-12770-s004.png]
